# The incidence of acute kidney injury in patients with traumatic brain injury

**DOI:** 10.1186/2197-425X-3-S1-A263

**Published:** 2015-10-01

**Authors:** R Avila, N Carrizo, A Fernandez, MM Filippi

**Affiliations:** Hospital Cullen, Santa Fe, Argentina

## Intr

There are a few researches that investigated non-neurological complications in traumatic brain injury (TBI) and reported a low incidence of acute kidney injury(AKI). Probably most of them did not use the scores validated for AKI ,likely only identify patients with severe AKI.

## Objectives

The aim of this study was to determine the incidence of AKI using a reliable score as classified by the RIFLE criteria.

## Methods

We study all patients, 16 years and over ,with moderate or severeTBI (Glasgow score < 13) and admitted to the intensive care unit (ICU) in a Trauma Intensive Care Unit Level III (Hospital Cullen) in Santa Fe, Argentina from 1 January 2014 to 31 December 2014.

Prospectively collected data of AKI from the ICU trauma registry and pathology database was analyzed retrospectively(Hardinero Quality). Risk injury failure loss end (RIFLE) criteria were used to categorize renal function.

## Results

The incidence of AKI was 17 % (29/169). RIFLE (F10,I5,R13) Patients who developed AKI were older, had higher severity of illness scores, and a lower GCS. ICU mortality : AKI group 58% compared with 30% in patients without AKI.

## Conclusions

In patients with TBI the incidente of AKI is relatively common and identifies patients with a high risk of death. Age, APACHE II and lower Glasgow score identify patients with high risk of AKI.
